# Effective Community-Based Interventions for the Prevention and Management of Heat-Related Illnesses: A Scoping Review

**DOI:** 10.3390/ijerph18168362

**Published:** 2021-08-07

**Authors:** Fariha Hasan, Shayan Marsia, Kajal Patel, Priyanka Agrawal, Junaid Abdul Razzak

**Affiliations:** 1 Department of Internal Medicine, Dow University of Health Sciences, MBBS, Karachi 74200, Pakistan; farihahassan100@gmail.com (F.H.); shayan.shoaib420@gmail.com (S.M.); 2 Center for Community Health, Northwestern University Feinberg School of Medicine, Chicago, IL 60611, USA; kajal.patel1@northwestern.edu; 3 Department of International Health, Johns Hopkins Bloomberg School of Public Health, Baltimore, MD 21205, USA; 4 Department of Emergency Medicine, Johns Hopkins School of Medicine, Baltimore, MD 21205, USA; junaid.razzak@jhu.edu

**Keywords:** heat wave, heat-related illnesses, urban settings, heat warning system

## Abstract

Background: Extreme temperatures have negative consequences on the environment, ecosystem, and human health. With recent increases in global temperatures, there has been a rise in the burden of heat-related illnesses, with a disproportionate impact on low- and middle-income countries. Effective population-level interventions are critical to a successful public health response. Objective: This scoping review aims to summarize the evidence on the effectiveness of population-level heat-related interventions and serve as a potential guide to the implementation of these interventions. Methods: Studies that evaluated the effectiveness of community-based interventions to mitigate or reduce the impact of extreme heat on heat-related mortality and morbidity were sought by searching four electronic databases. Studies published in the English language and those that had quantifiable, measurable mortality, morbidity or knowledge score outcomes were included. Results: The initial electronic search yielded 2324 articles, and 17 studies were included. Fourteen studies were based in high-income countries (HICs) (Europe, US, Canada) and discussed multiple versions of (1) heat action plans, which included but were not limited to establishing a heat monitoring system, informative campaigns, the mobilization of health care professionals, volunteers, social workers and trained caregivers in the surveillance and management of individuals with known vulnerabilities, or stand-alone (2) education and awareness campaigns. Multi-pronged heat action plans were highly effective in reducing heat-related mortality and morbidity, especially among vulnerable populations such as the elderly and those with chronic conditions. Conclusions: The heat action plans covered in these studies have shown promising results in reducing heat-related mortality and morbidity and have included instituting early warning systems, building local capacity to identify, prevent or treat and manage heat-related illnesses, and disseminating information. Nevertheless, they need to be cost-effective, easy to maintain, ideally should not rely on a mass effort from people and should be specifically structured to meet the local needs and resources of the community.

## 1. Introduction

Extreme temperatures are known to have negative consequences on the environment and the ecosystem [1]. Already more frequent and intense heat waves are likely to increase in the future due to a projected 0.1–0.2-degree Celsius rise in temperature by 2100 [2,3,4]. Extreme heat can lead to a spectrum of health-related conditions that range from mild to severe and include, but are not limited to, heat dehydration, cramps, exhaustion syncope and stroke; these are referred to as heat-related illnesses (HRIs) [5]. Without appropriate cooling strategies, extreme heat overextends the body’s capability to regulate its temperature, which can then lead to cardiovascular and/or respiratory compromise, multi-organ failure, impaired coagulation, loss of consciousness, stroke and even death [6].

The World Health Organization (WHO) estimates that 166,000 deaths have occurred from 1998–2017 due to heat-related illnesses [7,8,9,10]. The 2003 heat wave in Europe increased this number alone with an estimate of 70,000 deaths, while Russia saw 56,000 deaths in the heat wave of 2010 [11]. Other parts of the world have seen similar trends due to heat waves, especially in countries closer to the equator. These countries already experience higher temperatures at baseline, making them more likely to bear the impacts of even small increases in average temperature [12]. Countries in South Asia such as India and Pakistan have experienced heat waves that resulted in thousands of excess deaths [13,14].

Extreme temperatures have been shown to affect countries of all income levels; however, low- and middle-income countries (LMICs) are more vulnerable to the world’s worsening climate and are at increased risk of adverse outcomes for a variety of reasons [8]. LMICs have inadequate resources, expertise or focus to implement heat mitigation strategies. Fragile government institutions, overburdened community programs, weak emergency response, and hospital and medical delivery create additional barriers to long-term and consistent preparedness and response [12,15,16].

Upcoming research on extreme heat suggests that most fatalities associated with heat-related illnesses are preventable through strategies to warn and educate the public about HRIs, implement prevention programs and advocate for the implementation and enforcement of policies at population level to reduce the burden of HRIs [17,18,19]. However, most of these interventions have seen some fruition only in high-income countries, such as European countries, the United States and Canada.

The WHO and World Meteorological Organization (WMO) collaborated to produce a technical guide as an aid for governments to set up an early warning system for heat waves [20]. While governments in some HICs were able to implement these, other countries, especially those in low resource settings, have not been able to set up systems to mitigate the impacts of extreme heat. In areas where governments do not have resources to drive heat response, communities must make changes to their environment and behaviors to reduce the impact of extreme heat exposure.

To provide evidence on the effectiveness of these already-implemented interventions, we conducted a literature review to document the evidence around community-based interventions and summarize the impact of these interventions on mortality, morbidity and improved heat illness literacy among communities.

## 2. Materials and Methods

This scoping review was conducted in accordance with guidance of the Preferred Reporting Items for Systematic Reviews and Meta-Analyses Extension for Scoping Reviews [21].

### 2.1. Question and Study Eligibility

The main objective of the study was to identify community- and evidence-based interventions to manage heat-related illnesses in urban settings. Studies were deemed eligible if they provided explicit information on the effectiveness of community-based interventions implemented to address mortality, morbidity, or heat literacy on a population level. The PICO elements are listed in Table 1.

### 2.2. Inclusion Criteria for the Studies Were as Follows

(1) Original articles published in English.
(2) Studies employing randomized control trials, prospective, cross-sectional, or observational research methods to quantitatively evaluate the effectiveness of the interventions.
(3) Studies that conducted analyses of the cost-effectiveness of interventions.
(4) Interventions had to be population-level-based.
(5) Studies should have had full English translations available.

### 2.3. Exclusion Criteria

(1) Surveillance studies, literature reviews, reports, protocols, short communications, opinion pieces, case reports.
(2) Studies that described the epidemiologic profile of HRIs in different countries or regions.
(3) Studies that employed qualitative research methods.
(4) All sports and military exercise-related studies, including those focused solely on day laborers, hospital-based interventions, occupations, or those that addressed the impact of heat due to underlying co-morbidities.
(5) Studies on the impact of cold temperature on human health.

### 2.4. Study Search Strategy and Process

An electronic search was performed in November 2020 via databases including PubMed, EMBASE Global Health and WHO regional indexes. The study search strategy was determined after consulting with the librarian at the Welch Medical Library within the Johns Hopkins Medical Institutions, and the keywords used for the search were combinations of Medical Subject Headings (MeSH) terms for each database, respectively (Appendix A). Vocabulary and syntax were adjusted across databases. Publications were included from inception of the databases till November 2020. The search was restricted to studies in English, excluding experimental articles (ex vivo/in vitro/animal models) and others as per the inclusion and exclusion criteria. Records were collated in reference manager software (Endnote TM; Version: X7, Clarivate Analytics, New York, NY, USA) and transferred to a web-based platform, Covidence, that provides a systematic means of conducting reviews [22]. Titles of all records were screened independently by four reviewers and abstracts of the records were read to identify studies for further full-text reading. Any mismatch at this stage was resolved by discussion. Each of the selected full texts was similarly read, and suitability for inclusion was assessed by only one researcher and was verified independently by a reviewer.

### 2.5. Data Extraction and Outcome of Interest

Three reviewers extracted data from the studies included and tabulated this information using standardized templates built within Covidence. Data items included author title, year of publication, country and setting, study characteristics such as study design, population, sample size, details of the intervention, and findings from the study. The primary outcome of interest was the evidence-based quantifiable outcome measures (mortality, morbidity, KAP scores) from each study.

### 2.6. Quality Appraisal

As the final set of articles included in the review ranged from randomized controlled trails, time series analyses, observational studies, and pre-post intervention studies in terms of methodology, we applied the study quality assessment tools developed by the National Heart, Lung and Blood Institute to appraise the quality of the articles [23]. Tools specific for a methodology had 12 to 14 questions with items around clear objectives, defined population, sample size justification, clear definitions of exposure and outcome variables, among others. All questions had three responses, “Yes”, “No” and “Not applicable”. Four reviewers independently appraised randomly assigned articles and any mismatch was resolved via multiple team discussions. Articles were categorized as “Good” if 80% of all applicable responses were “Yes”, “Fair” if 40–80% of all applicable responses were “Yes” and “Poor” if less than 40% of all applicable responses were answered “Yes”.

## 3. Results

### 3.1. Study Selection

**Figure 1:**
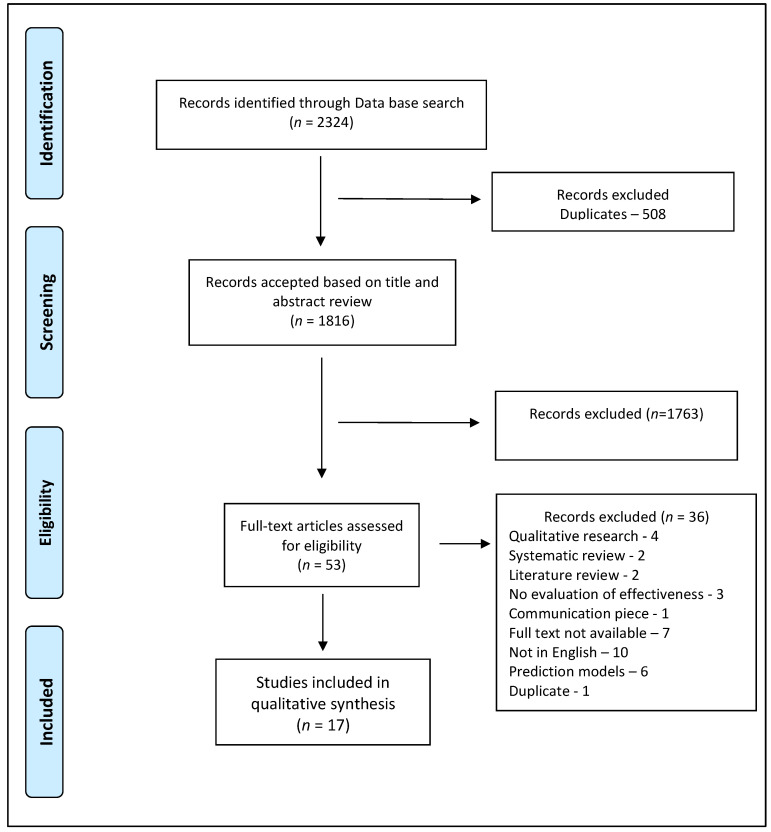
PRISMA flow chart highlighting the literature review.

Initially, there were a total of 2324 articles from all the four databases—PubMed, EMBASE, Global Health and WHO regional indexes. After removing 508 duplicates, 1816 articles were considered for title and abstract review. After abstracts were read, 1763 articles that were irrelevant based on the inclusion and exclusion criteria were removed. From these, 53 articles were considered suitable for full-text review. Thirty-six articles were excluded for reasons such as qualitative studies (*n* = 4), reviews (*n* = 4), full text not available (*n* = 7), not in English (*n* = 10), no evaluation of effectiveness or prediction models (*n* = 9), and duplicate/communication (*n* = 2). The remaining 17 articles met the inclusion criteria. The PRISMA flowchart exhibiting the study selection process is presented in Figure 1.

### 3.2. Study Characteristics

Of the 17 articles that were included in the final review, 14 articles were based in HICs, while three were based in LMIC settings. Most (10 out of 17) articles covered community-based interventions in the form of heat action plans and six were from Europe, which had been established by respective local and national governments in response to the 2003 heat wave. The studies ranged from randomized trials (*n* = 2), non-randomized or quasi-experimental analyses (*n* = 6) to observation or secondary data analyses. Variations in health outcomes reported, the assessment of knowledge, attitude and practices, sample populations, and data sources were observed (Table 2).

Results from the quality appraisal showed that 76.5% (*n* = 13) of all articles included were categorized as good, while the others were fair in nature (Table 1). All studies had clearly stated objectives and a defined population frame and sample. As most studies were observational in nature and applied secondary data analytical methods, the population frame was well established. However, only some studies justified their sample size and selection criteria. Articles that applied time series analyses were not able to define the underlying data collection and measurement techniques, and this reflects the pragmatic use of publicly available data from national governments, which cannot be controlled. As the data analytical methodology was strong and well defined, the findings from the study were considered reliable. It is important to note that for studies that applied randomized controlled trials to assess the effectiveness of an intervention or a package of interventions to address change in the knowledge of heat literacy, the interventions were subjective in nature and heavily relied on compliance from elderly participants—thus, the effectiveness of interventions in improving the outcome of knowledge was interpreted with caution.

The chosen articles elaborate on (1) the establishment of heat action plans and (2) education and awareness campaigns while accommodating age and need-appropriate dissemination of heat-specific preventive actions as effective interventions in reducing the burden of heat-related illnesses.

### 3.3. Heat Action Plans

Heat action plans were implemented mostly in high-income countries across Europe, in Canada and in Japan, and comprised of activities including, but not limited to, establishing a heat monitoring system, also known as the heat health watch warning system, informative campaigns for the general population, the mobilization of health care professionals, volunteers, social workers and trained caregivers in the surveillance and management of individuals with known vulnerabilities, as well as the provision of required infrastructure to cope with extreme temperatures. One study reported the implementation and evaluation of a heat action plan in a low- and middle-income country, India [24].

The PHASE project was implemented across multiple cities in Europe in response to the 2003 heat wave [25]. The project implemented a heat warning system and provided information to the general communities on preventive measures. Volunteers and social workers sensitized the communities. General physicians conducted surveillance by setting up a health information system for the identification of susceptible individuals and developed guidelines for specific health interventions for at-risk sub-groups, such as delaying discharge or postponing non-urgent surgical procedures [25]. The project also involved setting up emergency protocols in health centers and the activation of the national helpline during summers, which was managed by medical personnel and trained operators from the respective ministries of health [25]. Cities also opened cooling spaces. The helpline provided information on prevention tips for reducing health risks during heat waves.

The heat action plan implemented in France in 2006 focused its resources on isolated and vulnerable population groups and retirement homes and reported 4388 fewer heat-related deaths than expected based on historical trends [26]. A study reported the impact of the heat action plan under the PHASE project on various outcomes across multiple cities. There was a significant reduction in the attributable number of deaths in some cities [25]. Another study looking at the implementation of heat-action days in seven cities of France saw a combined reduction in the relative risk of mortality by 3.3% over a one-year period [27]. Similarly, the heat action plan implemented in Canada between 2000 and 2007 focused on providing reminders of preventive measures and daily contact via telephone or home visits to patients in hospitals and home care facilities [28]. The government distributed water bottles and made provisions for easy access to air-conditioned common spaces. The study found that implementing the heat action plan reduced daily deaths on average by 2.52 deaths per day in the 2004–2007 period compared to the 2000–2003 period [28]. Between the 2000 and 2007 period, the average daily deaths were around 39 and there were around 75 heat wave days during the same period. The reduction in mortality was greater for populations aged greater than 65 (reduction in mortality by 2.44 deaths per day during hot days compared to 0–65 years) and those from low socio-economic status (reduction in mortality by 2.48 deaths per day), but there was not much evidence that a significant difference was observed across genders.

A time series analysis conducted across 23 cities of Italy showed that the heat action plan was able to reduce the attributable fraction of heat-related deaths from 6.3% before the plan (1999–2002) was implemented to 4.1% after (2013–2016) [29]. Another study focusing on Florentine, Italy showed a general decrease in the odds of heat-related mortality among individuals 65 years and older, only when considering the apparent maximum temperature [30]. The apparent temperature is a measure of discomfort to humans due to hot conditions and is calculated as a combination of air temperature and relative humidity [30]. A time series analysis of 16 Italian cities focusing on the elderly population above 65 years showed a weaker relationship between heat and mortality in almost all cities after the heat action plan was implemented. When temperatures increased from 9 °C to 12 °C above the 25th percentile, the risk of mortality reduced from 36.75% in the pre-intervention phase to 13.31% post-intervention [31]. One intervention, “The Long Live the Elderly”, implemented in some urban areas of Italy, looked at countering social isolation among the elderly by maintaining frequent contact with the elderly over 75 years of age to carry out health promotion campaigns [32]. The study also aimed to strengthen the community network around sick and socially isolated individuals by involving people living or working near them in volunteer care actions. Compared to urban areas where the program was not being implemented, the urban areas with the LLE program showed a 13% reduction in heat-related mortality [32].

In another similar heat action plan implemented in Spain with the addition of a general hotline for emergency services, there was a small decrease in mortality attributable to extreme heat (from 0.67% (95% CI 0.61–0.72) to 0.56% (95% CI 0.52–0.59)), which was offset by an increase in mortality attributable to moderate heat (from 0.38% (95% CI 0.12–0.62) to 1.21% (95% CI 0.98–1.44)) [33]. The small reduction in the post-implementation phase was most prominent among those older than 85 years of age and in rural areas. Mild differences were observed by gender and socio-economic vulnerability of the town of residence [33].

A study conducted in four cities of North America looked at the impact of a heat mitigation plan among the elderly, aged 65 years and older [34]. Similar to heat action plans in Europe, these also comprised of a multi-institutional heat alert system (weather forecasters, media, city government, police and fire department, among others) and measures to prevent heat-related illnesses. In Dayton, guidelines on clothing, proper diet, keeping the house cool and not leaving the house were given out. A “buddy system” was executed in which local citizens were enlisted to check on vulnerable populations. In Philadelphia, an education program about heat-related illnesses was also carried out and senior centers were kept open for longer to provide support. Phoenix released comprehensive heat alerts by the National Weather Service office. Toronto executed training on heat-related illnesses and treatment for community agency staff and volunteers. A heat helpline, warning signs and the opening of cooling centers were some of the initiatives. Bottled water was handed out to the at-risk natives. Post-survey results indicated that knowledge of heat warnings was 90%. Surprisingly, behavioral modifications were less common at 46%. Even though the survey had multiple heat mitigation strategies, most people thought avoiding the outdoors was the best strategy. Air conditioning was considered the best method of cooling; however, its use was shown to be restricted due to costs.

Likewise, the heat action plan implemented in Ahmedabad, India created a similar multi-approach strategy—community awareness via outreach, health care capacity building, and setting up an early warning system [24]. Three levels of alerts were identified based on varying temperatures. Post-implementation, heat action plan warnings were associated with a decrease in all-cause mortality rates, with the largest decrease associated with extreme temperatures. An estimated total of 2380 deaths were avoided (95% Cl 324–4435). Short term evaluation of the heat action plan showed that the maximum relative risk at a temperature of 47 °C declined from 2.34 pre-intervention to 1.25 post-intervention.

### 3.4. Educational Interventions

Some studies conducted awareness sessions within the community settings that contained guidelines on preventing heat stress, providing information on high-risk population groups (vulnerable groups such as children and the elderly) and provisions for resources to use to prevent heat illness, among other topics, aiming to improve the community’s knowledge, attitudes and perceptions towards the prevention of heat stress. Like heat action plans, these studies were also administered in high- and middle-income countries such as China, the United States and Australia, but the medium used to disseminate the information differed from study to study, as highlighted below, with varying efficacy.

The Heat Wave Intervention Program (HWIP) in China, implemented in conjunction with the government, used a three-level “district, street and community” health care network. Community service operators were arranged to man a 24/7 hotline and WeChat to communicate with community members. Doctors were requested to share heat-mitigating information with patients who visited them for other reasons [35]. In Licheng district, additional government subsidies were provided, and provisions were made to be able to adjust work timings for the intervention group during high temperatures [35,36]. An increase in knowledge (β = 0.387, *p* < 0.001) and attitude (β = 0.166, *p* < 0.01) scores was seen in the post-intervention phase. Differences in the KAP scores were seen to be associated with age, gender, and marital status, among others.

Despite the low recall (25%) of the catchy slogan “Beat the Heat: Don’t forget your drink” in Australia, the use of unpaid advertisements on various media platforms led to the low reach of information dissemination [37]. However, participants self-rated their understanding of health risks at a 7.9 out of 10, and 54% of them attested to having changed their behavior over the summers [37]. Most studies targeting the elderly population used innovative and targeted approaches to facilitate knowledge uptake. A randomized study conducted in Southern Australia provided tangible material resources with messages such as health cards, fridge magnets, and fact sheets to intervention groups and found that a higher number of folks in the intervention group made the necessary lifestyle changes in terms of cooling methods (use of air conditioner and/or a wet cloth) over the summer [38]. Another study in the United States provided fridge magnets, critical temperature-marked thermometers with a hotline number, colored sheets, and one-on-one health education sessions to the elderly [39]. The proportion of elderly people who knew whom to contact for assistance rose significantly from 76% pre-intervention to 94% post-intervention. Another randomized community trial conducted in Japan included the door-to-door delivery of water bottles with short messages about heat prevention in addition to the community-wide dissemination of prevention strategies and was associated with increased water intake as well as reduced activity in the heat for the intervention groups [40].

## 4. Discussion

This review aimed to determine the effectiveness of community-based heat prevention programs in urban settings of both high- and low-income countries. Heat prevention programs were seen to focus on the development and implementation of heat action plans that required multi-sectoral engagement. The studies highlight the fact that local, regional, and national governmental agencies need to take ownership of heat action plans and lead other relevant institutions such as health care facilities, community homes, volunteer and social networks, among others, to manage multiple components of a multi-pronged heat action plan.

Most heat action plans included (1) instituting early warning systems, (2) building local capacity to identify, prevent or treat and manage heat-related illnesses, and (3) disseminating information. However, the interaction of individual-level and institutional-level barriers and facilitators within municipal organizations plays a humongous role in the uptake and successful implementation of such programs [41,42,43]. The existing heat action plans provide an excellent set of best practices for establishing successful plans in other settings. It would be safe to assume that plans that are locally led, collaborate with, and utilize local workforce and infrastructure resources and establish multi-lateral transparent communications to mitigate the impact of a heat wave would be more successful in resource-limited settings where financial constraints and large-scale environmental changes are a challenge [35,44].

Targeted programs and approaches for the vulnerable population—such as the distribution of water to the elderly via volunteers, mandating residents in hard-to-reach areas to have registered emails with local municipalities, and placing trigger systems in senior and retirement homes, schools, etc., providing an additional layer of vigilance and safety—are helpful in further reducing the impact of HRIs [45]. However, it is important to note that additional resources in the form of unpaid volunteers and infrastructure may be required to provide such interventions, which could become a barrier to implementation and widespread adoption, especially in resource-limited settings [46]. Heavy reliance on volunteers physically traveling and providing door to door information along with other free or low-cost provisions in reaching vulnerable people may pose accountability, safety, and heat-related challenges for volunteers as well.

The use of digital technology as a medium of information dissemination also has the potential to cast a wider net on reachability. However, only 40% of the LMIC population has mobile-cellular subscriptions, which is also the only mode of accessing the internet [47]. The use of mobile phones as a tool to promote physical activity or raise awareness about HIV has been shown to be effective in the past and has the potential for use as an early warning system and in raising awareness about heat-related illnesses [48]. As suggested in the articles, along with education, dissemination and setting up systems, it is important to build the capacity of local health care providers and collaborate with local leaders, religious leaders, and celebrities to influence community members in imbibing safe and precautionary practices. Culture and religion play a critical role in LMICs and can be a well-suited venue to help create behavior change among communities [49,50].

Another important aspect in the prevention of HRIs and successful heat prevention plans is the regular surveillance of variable temperatures throughout the year. Prior knowledge of impending extreme temperatures can facilitate the initiation of prevention strategies as well as early installment of programs such as relief camps. We encourage more collaboration of governments with the World Meteorological Organization to determine appropriate heat health warning systems to better classify and forecast heat emergencies on a more consistent and reliable basis [51].

There are some limitations to the study—as we focused on those articles that provided a change in a measurable health outcome or score due to the implementation of an intervention, the review might have missed other program-based studies that could provide implementation-related circumstantial evidence in the translation of interventions in other contexts. We also realized that the body of research regarding heat prevention programs from LMICs was limited, which limits the generalizability and feasibility of implementing action plans in limited-resource settings. Our search was also limited by language as only articles available in English were extracted.

While this review provides a menu of sorts on the packages of interventions that can be created to have a mitigating effect on the impact of extreme heat on human health, the lack of evidence around the effectiveness of these interventions in low-resource settings cannot be undermined. It is worthwhile to investigate the real-time impact of such interventions in low-resource settings as well as conduct studies to tease out the most beneficial package of interventions that are most effective, both in health outcomes and cost structures.

Our review also highlights several opportunities for strengthening evidence for heat intervention programs, specifically in low-resource settings, where there is an obvious research gap. There is a need to provide tried and tested strategies for individuals and families in settings where governments cannot or would not carry out population-level comprehensive programs or implement public health interventions.

Our review has also shown that research is heavily limited to pre-post intervention studies or secondary data analyses using publicly available national-level data. There is a need for better designed experimental studies that can control for uncontrollable differences such as temperature and humidity and controllable confounders such as access to and level of health care utilization and other social determinants of health.

Additionally, most studies have looked at the short-term impact of health interventions, and there is lack of evidence to gauge the long-term effectiveness of the same. While there is clear evidence to show that elderly people have benefited from the application of interventions, there is a need to assess the impact of interventions on other demographic groups of the population. It would also be interesting to see if the current set of tested interventions has any impact on the individual level of exposure to heat. Longer term studies to look at the change in this individual level of exposure are needed to further fill the gap.

Finally, given the difficulty in diagnosing heat-related mortality and morbidity due to the overlap in signs and symptoms across multiple organ systems, studies that can delineate the impact of interventions on direct heat mortality and morbidity are required.

## 5. Conclusions

For heat prevention plans to be implementable and successful, they need to be cost-effective, easy to maintain, ideally should not rely on a mass effort from people and should be specifically structured to meet the local needs and resources of the community. Most robust programs and their associated effectiveness as well as cost-effectiveness studies are needed, specifically in low-resource settings, to mitigate the effect of extreme heat conditions as well as understand the health and economic impacts of such interventions in the long term.

## Figures and Tables

**Table 1:** PICO elements used to frame the search strategy.

Criteria	Description
Population	Individuals living in urban settings across any country
Intervention	Community-based interventions to address heat-related illnesses
Comparator	Populations in different time periods, or without intervention
Outcome	Measurable outcomes such as excess mortality, attributable number of deaths, risk ratio, KAP (knowledge, attitude, practice) scores, and prevalence rate, which provide effectiveness of the intervention

**Table 2:** Summary of studies on community-based heat-related interventions in urban settings.

Author	Location	Study Design	Sample Population	Sample Size	Intervention Type	Primary Outcome	Comparator (If Any)	Quality	Results
Mattern 2000	United States	Cross-sectional study	Elderly above 65 years of age	34	Health education, culturally sensitive and age-specific heat-related manual	Risk factors for heat-related mortality	Same population before intervention	Good	67% (pre-test) versus 94% (post-test) knew of a contact for assistance during hot weather
Sheridan 2007	United States	Survey	Adults 65 years and above in four North American cities	908	Heat Mitigation Plan	Knowledge	NA	Good	Post-survey, knowledge—90% Behavior modification—46%
Fouillet 2008	France	Cohort study	Whole population of France	NA	Awareness; National Heat Wave Action Plan	Excess mortality	Same population before intervention	Good	Expected excess mortality ratio was +27% whereas observed excess mortality ratio was +9%, with an estimated mortality deficit of 2065 deaths
Oakman 2010	Australia	Observational study	All individuals above 18 years of age living in the area	328	Media awareness	Knowledge, attitude and practice	NA	Good	54% changed their summer behavior Self-rated understanding of the heat health risks at 7.9 on a 10-point scale, higher than same time last year
Morabito 2012	Italy	Cross-sectional study	Elderly above 65 years of age	21,092	Heat Health Warning System (HHWS)	Heat-related mortality	Same population before intervention	Good	Reduction in mortality rate observed only for 75 years and above, only when the maximum temperature time period was considered
Schifano 2012	Italy	Pre-post intervention study	Elderly above 65 years of age	50,000 to 2.5 million in the different cities	National heat health prevention program	Heat-related mortality	Same population before intervention	Good	Reduction in elderly mortality from +36.7% to +13.3% with increase in temperature from 9 °C to 12 °C above the 25th percentile
Pascal 2012	France	Statistical modeling	NA	~11 million	Heat warning system	Relative risk of mortality	NA	Good	Implementation of heat-action days was associated with a combined loss of relative risk of mortality by −3.3% (95% CI −10.3–4.4)
Takahashi 2015	Japan	Randomized controlled trial	Elderly 65 to 84 years of age	1072	Heat health warnings and distribution of water bottles	Knowledge, attitude and practice	No intervention group	Fair	Improvement in the frequency of water intake (*p* = 0.003) Improvement in frequency of cooling body (*p* = 0.002) Improvement in the frequency of taking a break (*p* = 0.088), Reduced activities in the heat (*p* = 0.093) Increase in hat or parasol use (*p* = 0.008)
de’Donato 2015	Europe	Quasi-Experimental	Deaths that occurred in 9 European cities	1,322,844	Heat Action Plan	Attributable number of deaths	Same population before intervention	Good	In terms of heat attributable mortality, 985, 787 and 623 fewer deaths estimated in Athens, Rome and Paris, respectively. A reduction in mortality risk associated with heat observed only in the three aforementioned cities.
Benmarhnia 2016	Canada	Quasi-Experimental	All residents of the island of Montreal	NA	Advisories and emergency public health measures	Heat-related mortality	NA	Good	Daily deaths reduced by an average of 2.52 deaths per day after implementation of the heat action plan
Nitschke 2017	Australia	Randomized controlled trial	Elderly above 65 years of age	637	Awareness; Evidence-based information leaflets	Behavior	No intervention group	Good	Intervention group had significant increases in: air conditioner use during hot weather (74.4% versus 63.4%) the use of a wet cloth on face, neck or body to cool down during heat waves (16% vs. 8%) the belief that they had enough information to beat the heat (94% vs. 88%)
Hess 2018	India	Time series analysis	People living in Ahmedabad city	Entire population	Awareness and Health Intervention, Heat Action Plan (HAP)	Risk ratio	Pre intervention period, same population	Good	Post-to-pre-HAP non-lagged mortality IRR for maximum temperature over 40C was 0.95 (0.73–1.22) and 0.73 (0.29–1.81) for maximum temperature over 45C. An estimated 2380 deaths post-intervention were avoided
Xu 2018	China	Quasi experimental	All individuals above 14 years of age living in the area	2400	Health care networks	Knowledge, attitude and practice	No intervention group	Fair	Intervention groups had 0.387, 0.166 and 0.037 higher knowledge, attitude and practice scores, respectively
de’ Donato 2018	Italy	Time series analysis	People residing in 23 Italian cities	NA	Awareness and Health Intervention;Italian National Heat Plan	Attributable number of deaths	NA	Fair	For extreme temperatures. The attributable fraction of heat-related deaths declined from 6.3% in the period 1999–2002 to 4.1% in 2013–2016. More than 1500 heat attributable deaths spared
Liotta 2018	Italy	Non-randomized experimental study	Elderly above 75 years of age	12,207	Social Intervention:The Long Live the Elderly (LLE) program to counteract social isolation	Heat-related mortality	No LLE urban areas	Good	Cumulative mortality rates of 25% (Cl 95%: 23–29) and 29% (Cl 95%: 17–43) in LLE versus non LLE urban areas, respectively
Martinez-Solanas 2019	Spain	Time series analysis	People living in Spain	NA	Prevention Plan;Spain’s National Heat-Health Prevention Plan (HHPP)	Attributable number of deaths	Same population, pre-intervention	Good	There was a small decrease in mortality attributable to extreme heat (from 0.67% to 0.56%), which was offset by an increase in mortality attributable to moderate heat (from 0.38% to 1.21%). Most significant reduction seen among older individuals.
Scortichini 2018	Italy	Time series analysis	Residents in 23 Italian cities	NA	National heat health warning system. Time mortality surveillance systemIdentification of susceptible individuals and treatment	Mortality rateAttributable number of deaths	Same population, pre-intervention	Fair	The effect of extreme temperature reduced after all cities implemented the heat action plan (RR 1.23, 95% 1.15–1.32). Attributable number of deaths reduced from 6.3% to 4.1% (1200 units) during periods of extreme temperature

## Data Availability

Not applicable.

## Appendix A

**Table A1:** Search strategy for the four data bases included in the literature review.

Database	Search Strategy
PubMed	"hot temperature"[MeSH Terms] OR "extreme heat"[MeSH Terms] OR "heat wave*"[Title/Abstract] OR "heatwave*"[Title/Abstract] OR "extreme heat"[Title/Abstract] OR "hot temperature*"[Title/Abstract] OR "hot weather"[Title/Abstract] OR "extreme weather"[Title/Abstract] OR "heat event*"[Title/Abstract] OR "Heat Stress disorders"[MeSH Terms] OR "Heat Stroke"[MeSH Terms] OR "Heat Exhaustion"[MeSH Terms] OR "sunstroke*"[Title/Abstract] OR "sun stroke*"[Title/Abstract] OR (("heat"[Title/Abstract] OR "heat-related"[Title/Abstract]) AND ("stroke*"[Title/Abstract] OR "illness*"[Title/Abstract] OR disorder*[Title/abstract] OR "exhaustion"[Title/Abstract] OR "prostration"[Title/Abstract] OR "edema*"[Title/Abstract] OR "oedema*"[Title/Abstract] OR "injur*"[Title/Abstract])) AND "heat health"[Title/Abstract] OR "health knowledge, attitudes, practice"[MeSH Terms] OR "Health Literacy"[MeSH Terms] OR "community-based participatory research"[MeSH Terms] OR "Health Literacy"[Title/Abstract] OR ("heat"[Title/Abstract] AND "literacy"[Title/Abstract]) OR ("heat"[Title/Abstract] AND "knowledge"[Title/Abstract]) OR ("heat"[Title/Abstract] AND "awareness"[Title/Abstract]) OR "community based"[Text Word] OR "community placed"[Title/Abstract] OR "action plan*"[Title/Abstract] OR "prevention plan*"[Title/Abstract] OR "training program*"[Title/Abstract]
Embase	’extreme hot weather’/exp OR ’heat wave’/exp OR ’high temperature’/exp OR ’heat stress’/exp OR ’heat injury’/exp OR ’heat wave*’:ti,ab,kw,de OR heatwave*:ti,ab,kw,de OR sunstroke*:ab,ti,de,kw OR ’sun stroke*’:ab,ti,de,kw OR ’extreme heat’:ti,ab,kw,de OR ’extreme weather’:ti,ab,kw,de OR ’hot weather’:ti,ab,kw,de OR ’hot temperature*’:ti,ab,kw,de OR ’heat event*’:ti,ab,kw,de OR ((heat NEAR/5 stroke*):ab,ti,de,kw) OR ((’heat related’ NEAR/5 stroke*):ab,ti,de,kw) OR ((heat NEAR/5 illness*):ab,ti,de,kw) OR ((’heat related’ NEAR/5 illness*):ab,ti,de,kw) OR ((heat NEAR/5 exhaustion):ab,ti,de,kw) OR ((’heat related’ NEAR/5 exhaustion):ab,ti,de,kw) OR ((heat NEAR/5 prostration):ab,ti,de,kw) OR ((’heat related’ NEAR/5 prostration):ab,ti,de,kw) OR ((heat NEAR/5 edema*):ab,ti,de,kw) OR ((’heat related’ NEAR/5 edema*):ab,ti,de,kw) OR ((heat NEAR/5 oedema*):ab,ti,de,kw) OR ((’heat related’ NEAR/5 oedema*):ab,ti,de,kw) OR ((heat NEAR/5 injur*):ab,ti,de,kw) OR ((’heat related’ NEAR/5 injur*):ab,ti,de,kw) OR ((heat NEAR/5 syncope):ab,ti,de,kw) OR ((’heat related’ NEAR/5 syncope):ab,ti,de,kw) AND ’heat health’:ti,ab,kw,de OR ’attitude to health’/exp OR ’health literacy’/exp OR ’participatory research’/exp OR ’participatory research’:ti,ab,kw,de OR ’community based’:ab,kw,ti,de OR ’community placed’:ab,kw,ti,de OR ’health literacy’:ti,ab,kw,de OR ((heat NEAR/5 awareness):ab,ti,de,kw) OR ((heat NEAR/5 knowledge):ab,ti,de,kw) OR ((heat NEAR/5 literacy):ab,ti,de,kw) OR ’action plan*’:ab,kw,ti,de OR ’prevention plan*’:ab,kw,ti,de OR ’training program*’:ab,kw,ti,de
Global Health	"heat wave*".mp. OR "heatwave*".mp. OR "extreme weather".mp. OR "extreme heat".mp. OR "hot weather".mp. OR "heat event*".mp. OR "sunstroke*".mp. OR "sun stroke*".mp. OR (("heat".mp. OR "heat-related".mp.) AND ("stroke*".mp. OR "illness*".mp. OR disorder*.mp. OR "exhaustion".mp. OR "prostration".mp. OR "edema*".mp. OR "oedema*".mp. OR injur*.mp.)) AND ("heat health" or "attitude to health" or "health literacy" or "participatory research" or "community based" or "community placed" or "heat awareness" or "awareness of heat" or "heat knowledge" or "knowledge of heat" or "heat literacy" or "action plan*" or "prevention plan*" or "training program*").mp
WHO Regional Indexes	**First concept:** Extreme Heat (no narrower terms): G16.500.750.775.271, N06.230.300.100.725.232.500, N06.230.300.100.725.710.380.500 Heat stress = Heat-Shock Response (no explode) G07.775.500 Heat Stress Disorders (with explode to include Heat Exhaustion, Heat Stroke, and sunstroke (narrower under heat stroke)) C26.522 **Second concept:** Health Knowledge, Attitudes, Practice F01.100.150.500, N05.300.150.410 Health Literacy I02.233.332.186.500, L01.143.450.500, N02.421.726.407.229.500 Community Based Participatory Research H01.770.644.193, N05.425.104 Attitude to Health F01.100.150, N05.300.150 **Search String:** G16.500.750.775.271 OR N06.230.300.100.725.232.500 OR N06.230.300.100.725.710.380.500 OR G07.775.500 OR C26.522$ AND F01.100.150.500 OR N05.300.150.410 OR I02.233.332.186.500 OR L01.143.450.500 OR N02.421.726.407.229.500 OR H01.770.644.193 OR N05.425.104 OR F01.100.150 OR N05.300.150
